# A Rapid Diagnosis and Treatment of a Traumatic Aortic Transection: A Case of Survival to the ICU

**DOI:** 10.7759/cureus.12726

**Published:** 2021-01-15

**Authors:** Larissa Dub, Sherwin Z Thomas, Nicholas Fusco, Cherian I Plamoottil, Latha Ganti

**Affiliations:** 1 Emergency Medicine, University of Central Florida College of Medicine, Orlando, USA; 2 Emergency Medicine, Osceola Regional Medical Center, Kissimmee, USA; 3 Emergency Medicine, Osceola Regional Medical Center, Orlando, USA; 4 Emergency Medicine, Envision Physician Services, Plantation, USA; 5 Emergency Medicine, Ocala Regional Medical Center, Kissimmee, USA; 6 Emergency Medicine, HCA Healthcare Graduate Medical Education Consortium Emergency Medicine Residency Program of Greater Orlando, Orlando, USA

**Keywords:** major trauma, aortic injury, emergency medicine and trauma, ultrasound anatomy, general thoracic surgery, motor vehicle collison

## Abstract

We present the case of a young man with traumatic aortic dissection secondary to a motor vehicle collision. While the differential diagnosis for traumatic injury after a motor vehicle collision can include commonly studied and trained for cases, such as pneumo/hemothorax, pulmonary contusion, splenic laceration, and pelvic fractures, for example, one of the more deadly and hence rare presentations of motor vehicle trauma is aortic transection. The fact that the diagnostic studies included as part of the initial Advanced Trauma Life Support^®^ (ATLS^®^) trauma survey are not well equipped to diagnose such an injury is also a deadly factor. In this case review, we explore factors affecting the timely diagnosis, management, and outcomes of traumatic aortic injury. Prompt diagnosis is imperative in order to save a patient's life.

## Introduction

Trauma secondary to motor vehicle accidents is not an uncommon presentation to the emergency department as it accounts for approximately 10% of ED visits [1]. Traumatic aortic injury is a rather uncommon presentation, accounting for less than 1% of ED visits [2]. After intracranial hemorrhage, blunt aortic injuries are the second most common cause of death in a motor vehicle collision. Of note, 81% of blunt aortic injuries are caused by motor vehicle collisions. However, only 20% of patients who sustain a blunt aortic injury even make it to the emergency department [3]. The majority of patients who sustain these injuries will succumb to their injury on scene. Prompt recognition and treatment are critical for patient survival. Aortic injury needs to be considered and diagnosed quickly upon initial evaluation within the emergency department. Several modalities can be considered such as ultrasound and X-ray/CT imaging. However, time is of the essence. The authors of this case report will present a case of a traumatic aortic transection resulting from a motor vehicle collision in which prompt diagnosis with ultrasound contributed to the patient’s survival. 

## Case presentation

**Figure 1:**
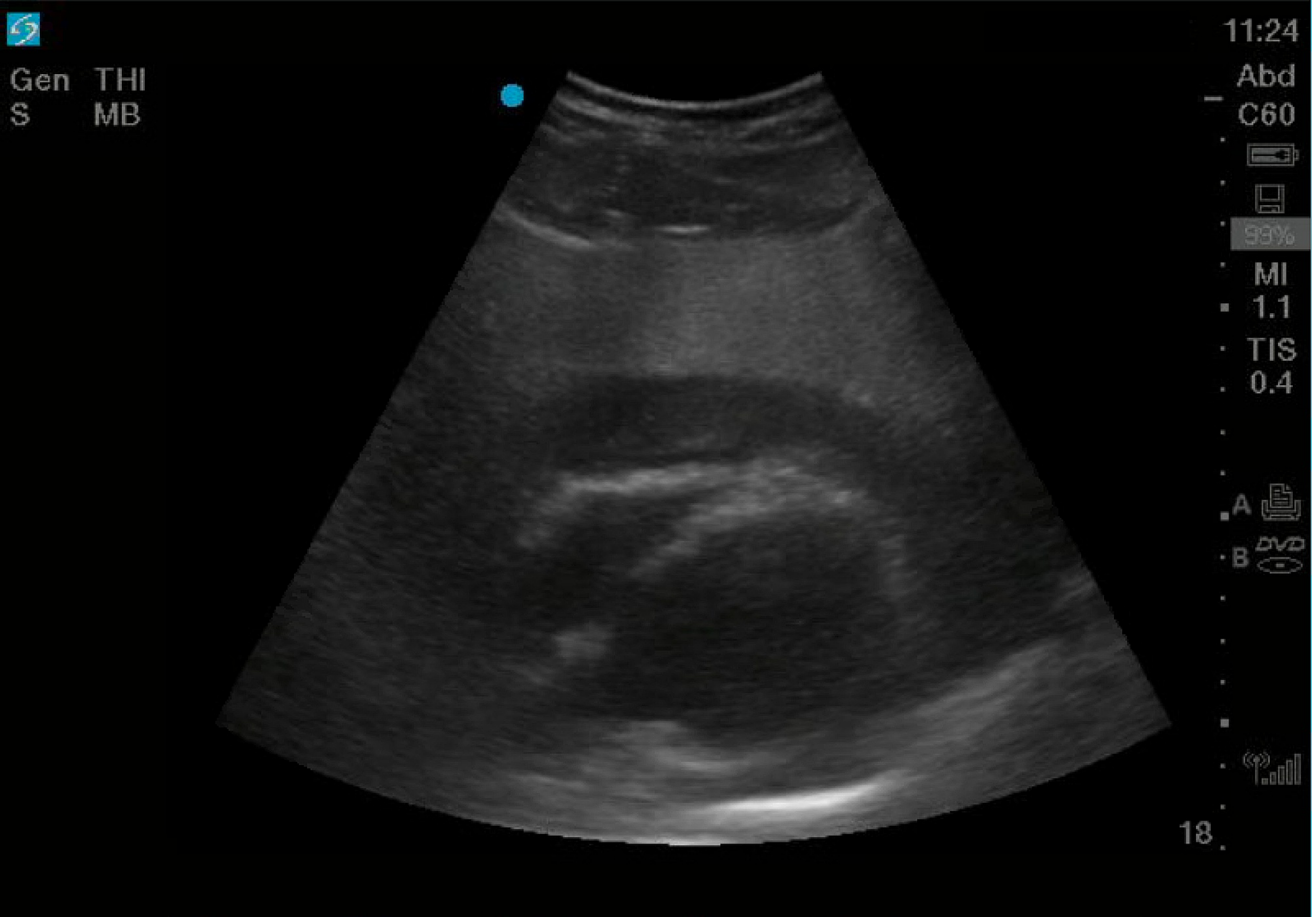
Ultrasound demonstrating a large pericardial effusion

**Figure 2:**
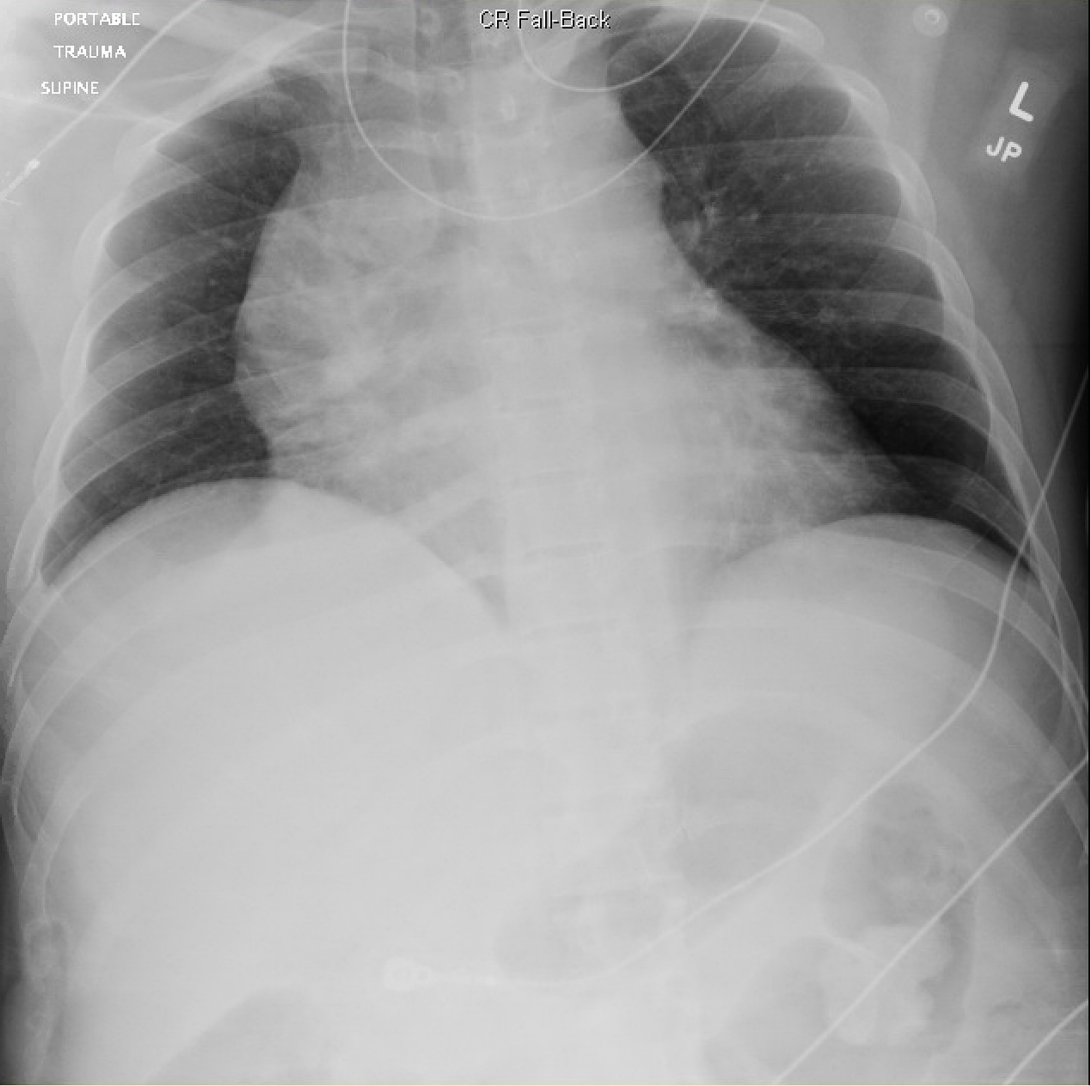
CXR with widened mediastinum

During a typical chaotic day in the emergency room, a prehospital trauma alert notification was called in over the radio. Per Emergency Medical Service (EMS), an approximately 30-year-old male was involved in a single-vehicle roll over motor vehicle collision. The patient self-extricated from the vehicle and was found lying outside the vehicle upon EMS arrival. On scene, the patient had a GCS of 15 and was only complaining of upper mid-back pain and right knee pain. It is unknown if the patient was restrained. His vitals were reported as “normal.” In route, the patient began to become more somnolent. Upon arrival to the emergency room, the patient was immediately placed on a monitor and full Advanced Trauma Life Support® (ATLS®) evaluation began. The patient was alert and answering questions, but appeared pale and diaphoretic. His only complaint was mid-back pain. His vitals were blood pressure (BP) 121/87, heart rate 98, respiratory rate 13, oxygen saturation (SpO_2_) 98%. During initial evaluation, a bedside point-of-care ultrasound (POCUS) was performed. Upon cardiac evaluation, the patient was found to have a large pericardial effusion (Figure 1). The remainder of his focused assessment with sonography in trauma (FAST) was unremarkable. A bedside Chest X-ray (CXR) was obtained that demonstrated an enlarged mediastinum (Figure 2). The patients’ blood pressure continued to decline and his last recorded systolic blood pressure systolic in the emergency room was 90. The decision to initiate our massive transfusion protocol was made and the patient began receiving packed red blood cells (PRBCs) in the emergency room. After consultation with the trauma surgeon, vascular surgeon, and cardiothoracic surgeon, the patient was then quickly taken to the operating room for probable thoracotomy.

Upon arrival to the operating room, the patient was intubated and bedside transesophageal echocardiogram (TEE) was performed that demonstrated a large pericardial effusion. An aortogram was performed by the vascular surgeon and the patient was found to have a mid-thoracic transection. Following thoracic endovascular aortic repair (TEVAR) repair, the patient remained hemodynamically unstable, so a decision to proceed with median sternotomy was undertaken. The patient was found to also have a laceration of his right atrium and inferior vena cava (IVC). These were subsequently repaired and the patient was taken to the intensive care unit (ICU) for further care and treatment. 

## Discussion

The clinical presentation of blunt aortic rupture is very nonspecific and encompasses symptoms and findings that match all types of traumatic injuries, not just aortic transection. Symptoms may include chest pain, back pain, abdominal pain or shortness of breath. Physical exam findings that may contribute to an increased index of suspicion include seatbelt or steering wheel signs on the chest wall [4]. While laboratory studies are a part of ATLS protocol, they are not useful in the diagnosis of aortic injuries. Imaging modalities that can be used are chest x-ray (CXR), transthoracic echocardiography (TTE), transesophageal echocardiography (TEE), ultrasound, and contrast-enhanced computed tomographic angiography (CTA). CXR and ultrasound are obtained as part of the ATLS algorithm for all trauma patients. CXR is non-diagnostic, but can demonstrate a widened mediastinum (as demonstrated in our patient in Figure 1), displacement of the left mainstem bronchus, or deviation of the trachea to the right [5]. The use of bedside ultrasound in the assessment of trauma patients has become the standard of care in the initial phases of trauma resuscitation. Proficiency in ultrasound has increased with advances in physician training and technology, making ultrasound an easily accessible modality to use at the bedside to make rapid clinical decisions and decrease time to diagnosis of critical conditions. In general, the ability of emergency physicians to detect pericardial effusions in trauma is very accurate. The use of bedside ultrasound to detect effusion has been shown to have sensitivities of up to 96% and specificities of up to 98% for the above etiologies. TTE is very poor at visualizing aortic trauma, however, it can point to possible signs of aortic injury such as the visualization of left hemothorax or pericardial effusion/tamponade [6]. TEE and CTA are the most useful and are both equal in the effectiveness of actually diagnosing blunt aortic injury [7]. The use of each modality is dependent on the hemodynamic stability of the patient and institutional limitations. 

## Conclusions

There are no studies evaluating the time window for possible salvage and survival of a blunt aortic injury. Given the high mortality rate in such patients, immediate intervention is the obvious choice. Whatever the route, the key to survival rests on the ability of the emergency medicine physician to make this initial diagnosis and involve specialists as quickly as possible.
